# A process evaluation of a cluster randomised trial to reduce potentially inappropriate prescribing in older people in primary care (OPTI-SCRIPT study)

**DOI:** 10.1186/s13063-016-1513-z

**Published:** 2016-08-03

**Authors:** Barbara Clyne, Janine A. Cooper, Carmel M. Hughes, Tom Fahey, Susan M. Smith, on behalf of the OPTI-SCRIPT study team

**Affiliations:** 1 Department of General Practice, HRB Centre for Primary Care Research, Royal College of Surgeons in Ireland (RCSI), 123 St. Stephens Green, Dublin 2, Republic of Ireland; 2 School of Pharmacy, Queen’s University Belfast (QUB), 97 Lisburn Road, Belfast, BT9 7BL Northern Ireland

**Keywords:** Potentially inappropriate prescribing, Process evaluation, Cluster randomised controlled trial, Primary care

## Abstract

**Background:**

The OPTI-SCRIPT cluster randomised controlled trial (RCT) found that a three-phase multifaceted intervention including academic detailing with a pharmacist, GP-led medicines reviews, supported by web-based pharmaceutical treatment algorithms, and tailored patient information leaflets, was effective in reducing potentially inappropriate prescribing (PIP) in Irish primary care. We report a process evaluation exploring the implementation of the intervention, the experiences of those participating in the study and lessons for future implementation.

**Methods:**

The OPTI-SCRIPT trial included 21 GP practices and 196 patients. The process evaluation used mixed methods. Quantitative data were collected from all GP practices and semi-structured interviews were conducted with GPs from intervention and control groups, and a purposive sample of patients from the intervention group. All interviews were transcribed verbatim and analysed using a thematic analysis.

**Results:**

Despite receiving a standardised academic detailing session, intervention delivery varied among GP practices. Just over 70 % of practices completed medicines review as recommended with the patient present. Only single-handed practices conducted reviews without patients present, highlighting the influence of practice characteristics and resources on variation. Medications were more likely to be completely stopped or switched to another more appropriate medication when reviews were conducted with patients present. The patient information leaflets were not used by any of the intervention practices. Both GP (32 %) and patient (40 %) recruitment rates were modest. For those who did participate, overall, the experience was positively viewed, with GPs and patients referring to the value of medication reviews to improve prescribing and reduce unnecessary medications. Lack of time in busy GP practices and remuneration were identified as organisational barriers to future implementation.

**Conclusions:**

The OPTI-SCRIPT intervention was positively viewed by both GPs and patients, both of whom valued the study’s objectives. Patient information leaflets were not a successful component of the intervention. Academic detailing and medication reviews are important components in changing PIP, and having patients present during the review process seems to be a more effective approach for decreasing PIP.

**Trial registration:**

Current controlled trials ISRCTN41694007. Registered on 21 March 2012.

## Background

Potentially inappropriate prescribing (PIP) comprises a number of suboptimal prescribing practices, including inappropriate dose or duration of medication, drug–drug interactions, drug–disease interactions, and use of medications that have a significant risk of an adverse drug event (ADE) [1]. Recent systematic reviews report an estimated PIP prevalence of 20 % in community-dwelling patients [2, 3]. PIP has become an important public health concern as patients with PIP have been found to have a more than twofold increased odds of experiencing adverse drug reactions [4] and a nearly a twofold increased risk in the expected rate of emergency room visits [5]. PIP is also associated with increased health expenditure [6, 7].

Decreasing the prevalence of PIP may have important public health and financial benefits, particularly in primary care, where the majority of prescribing occurs. However, to date, no single interventional strategy has proven to be most effective [8–11].

The OPTImizing PreSCRIbing for Older People in Primary Care, a clusTer randomised controlled trial (OPTI-SCRIPT) cluster randomised controlled trial (RCT) demonstrated that a multifaceted intervention was effective in reducing PIP in primary care. The intervention worked principally on reducing proton pump inhibitor prescribing and appeared less effective on other classes of PIP drugs such as long-term benzodiazepine prescribing and therapeutic duplication [12]. The detailed methods and results have been published elsewhere [12–14]. In brief, the intervention involved academic detailing with a pharmacist on conducting General Practitioner (GP)-led medicines review with participating patients; medicines reviews were supported by web-based pharmaceutical treatment algorithms for GPs, providing evidence-based alternative treatment options to PIP drugs, and tailored patient information leaflets (PILs). A summary of the study is presented in Appendix 1.

Process evaluations are recommended to contextualise RCT results, answering key questions about why an intervention has failed or succeeded and how it was implemented [15]. Process evaluations are particularly relevant to complex, multifaceted interventions such as OPTI-SCRIPT. Such interventions involve multiple targets (for example, patients and clinicians) and various active components and are often criticised as their complexity makes it difficult to measure their effects [16, 17]. To date, systematic guidance on what data a process evaluation should collect and report has been lacking and process evaluations have been planned and conducted in an ad hoc fashion [18]. Grant et al., recently developed a framework to guide the design and conduct of process evaluations specifically for cluster RCTs [19]. The framework presents a range of approaches to understanding trial delivery, intervention implementation, and the responses of targeted participants, taking into consideration evaluation at both the cluster and the individual participant level.

This paper presents the process evaluation conducted as part of the OPTI-SCRIPT trial and aims to explore how the intervention was implemented, the experiences of those participating in the study and lessons for future implementation.

## Methods

The process evaluation undertaken used a mixed methods approach, combining both qualitative and quantitative data. The process evaluation framework outlined by Grant et al. [19] was used to report the overall findings.

This study was approved by the research ethics committee of the Irish College of General Practitioners (ICGP) and informed consent was given by all GP practices and patients.

### Study population

**Fig. 1:**
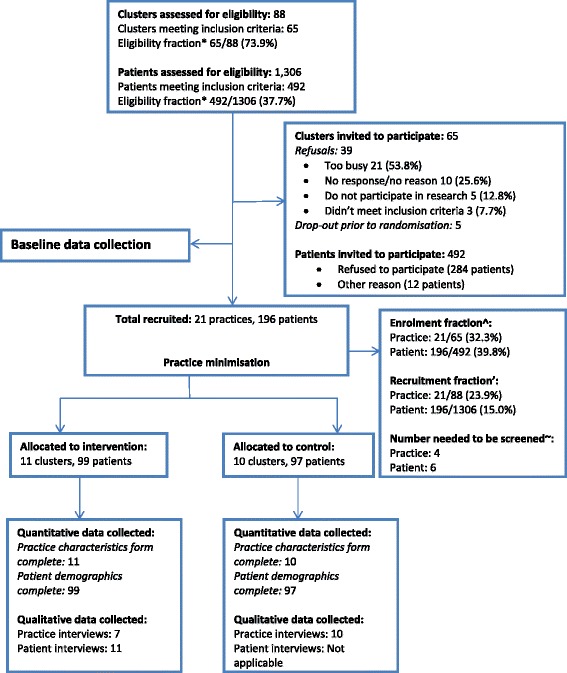
Flow of practices and patients through the study. *Eligibility fraction: Proportion of potential participants who undergo screening and are eligible to enrol. ‘Recruitment fraction: Proportion of potential participants who actually enrol in the RCT. ^Enrolment fraction: Proportion of people who are eligible for participation and who actually enrol in the RCT. ~Number of patients needed to be screened: Number of patients screened in order to randomise one participant (1/recruitment fraction). Source: adapted from Gross et al. 2002 [50]

The OPTI-SCRIPT study was conducted in the Irish primary care setting (see Appendix 2) among patients aged 70 years and over as the prevalence of PIP is high in this age group (36 % in 2007 [20]). Out of 21 GP practices, 11 practices (99 patients) were allocated to the intervention group and 10 practices (97 patients) were allocated to the control group using minimisation (Fig. 1).

### Qualitative sampling

The lead GP in all of the 21 GP practices was asked to participate in qualitative interviews with 17 agreeing, 13 (76.4 %) of whom were male. For patient interviews, only those who experienced the intervention were invited to participate (as questions mainly related to the experience of intervention). Purposive sampling was used to ensure coverage across GP practices and heterogeneity in terms of patient gender, type of PIP drugs, outcome of medication review. Out of 14 patients approached in the intervention group, 11 patients agreed to participate in an interview. Fifty-four percent of patient interviewees were male compared to 53.5 % of non-interviewees. The average age of interviewees was 78 years compared to 77 for non-interviewees.

### Data collection

Quantitative data were compiled from a number of sources. First, all GP practices completed a practice characteristics form upon recruitment, providing details on numbers of staff, location and numbers of patients. Second, all participating patients completed a questionnaire providing basic demographic details, health service utilisation and patient-reported outcome measures. Third, the OPTI-SCRIPT study team maintained researcher logs of the recruitment process and all contacts with participants. Finally, in the intervention group, process measure data were collected by evaluation forms, which were integrated into the web-based pharmaceutical treatment algorithms. These forms were completed by GPs upon completion of each OPTI-SCRIPT medication review.

Qualitative data were collected using semi-structured interviews. Intervention group patients (n = 11) were interviewed within 1 month of undertaking the OPTI-SCRIPT medicines review and intervention group GPs (n = 7) were interviewed within 1 month of completion of the final OPTI-SCRIPT medicines review for that practice. Control group GPs (n = 10) were interviewed upon final data collection conducted at intervention completion. Interviews were conducted by a single interviewer (BC) either in person or via telephone. Telephone interviewing is generally used where time or costs are issues, and evidence suggests there is little difference in the answers obtained this way [21, 22]. All interviews were audio recorded (on loud speaker for telephone interviews). The semi-structured GP interview topic guide was used to facilitate discussion of common prescribing-related issues in older patients and experiences of trial participation such as problems with the study processes or intervention and improvements which could be made. The patient topic guide focused on patients’ perceptions of their medications and their experiences of the medication review process (Appendix 3). Patient interviews lasted an average of 11.6 minutes (min 6.2–max 20.3), while GP interviews lasted an average of 14.5 minutes (min 8.56–max 26.31).

### Data analysis

Quantitative data were inputted into STATA Version 13 (StataCorp, College Station, TX, USA) and summarised using descriptive statistics.

All interviews were audiotaped and transcribed verbatim. A thematic analysis was performed using a ‘top down’ approach to coding [23], using the pre-defined categories of the Grant et al. framework as overarching themes [19]. Familiarisation with data was achieved through reading and re-reading transcripts one by one in detail. Field notes and observations were not used in the analysis. Initial codes were generated from commonly occurring patterns and were grouped into potential sub-themes and related directly to the pre-determined framework. Quotations were used as exemplars of key themes.

NVivo 10 was used to assist with organising the data for analysis. All transcripts were reviewed independently by two researchers (BC, JAC) and the findings discussed to confirm the validity of the emerging results. All participant data were pseudo-anonymized by assignment of a unique study ID.

## Results

The results are summarised in Table 1.

**Table 1:** Summary of methods and findings of the OPTI-SCRIPT process evaluation

Domain	Research focus	Data source	Main findings
Recruitment of practices	How were practices sampled and recruited? Reasons for non-participation?	Study team recruitment logs	Practices recruited from the HRB primary care research network by email with follow-up call. Recruitment modest, main reason for declining was practice being too busy.
Delivery to practices	What intervention is delivered for each practice? Is it the one intended by the researchers?	Semi-structured interviews	Academic detailing delivered to intervention practices as planned. Letters sent to control practices as planned.
Response of practices	How is the intervention adopted by clusters?	Website activity, semi-structured interviews	Medication reviews conducted as planned by eight (73 %) intervention practices, two (18 %) conducted reviews without patients present. Two (20 %) control practices made changes to patients.
Recruitment and reach in individuals	Who actually receives the intervention in each setting? Are they representative?	Study team recruitment logs, patient questionnaire data	Patients recruited broadly similar to national population demographically.
Delivery to individuals	Who received medication reviews?	Semi-structured interviews, website activity	Eighty-six patients had reviews, one practice conducted no reviews.
	How were reviews conducted?	Semi-structured interviews	Eight intervention practices conducted reviews with patients, two practices conducted reviews without patients present.
	What were the outcomes of the reviews?	Website activity	Most common outcome – dose reduction.
Responses of individuals	How does the target population respond?	Semi-structured interviews	Patients happy to participate and valued the opportunity to review unnecessary medication.

Source: adapted from Grant et al. 2013 [19]

*Abbreviations: OPTI-SCRIPT* OPTImizing PreSCRIbing for Older People in Primary Care, a cluster randomised controlled trial, *HRB* Health Research Board

### Recruitment of practices

A total of 65 eligible GP practices from 88 in the HRB Centre for Primary Care Research practice network were invited (by email with follow-up phone call) to participate. No national register of GPs exists in Ireland so this network offered a convenient sample of GPs to contact. The area was restricted to greater Dublin to facilitate the academic detailing process. Initially, 26 practices agreed to participate but five practices withdrew prior to patient recruitment^1^ and practice randomisation, due to time constraints, meaning a total of 21 practices (32.3 %) participated (Fig. 1). Of the 39 practices who declined to participate, the majority (54 %) did so as the practice was too busy (Fig. 1). In contrast to a national sample (Table 2), participating study practices were slighter larger in terms of the average number of GPs and General Medical Services (GMS) patient lists, and were all involved in undergraduate/postgraduate teaching.

**Table 2:** Comparison of OPTI-SCRIPT participating practices and patients to national populations

Characteristic	Study participants	National population
GP practice		
Practice type		
GMS and private	100 %	96.0 %^a^
GMS list size		
500 or less	14.3 %	29.8 %^b^
501–1500	23.8 %	59.6 %^b^
1501–2500	61.9 %	10.6 %^b^
Practice staff		
Single-handed GP	14.3 %	35.0 %^a^
2+ GPs	85.7 %	65.0 %^a^
Practice manager	71.4 %	30.0 %^a^
Practice location		
Urban	76.2 %	43.0 %^a^
Mixed	23.8 %	36.0 %^a^
Teaching activity	100 %	42.0 %^a^
Patients		
Total population		
Male	105 (53.6 %)	157,016 (43.4 %)^c^
Age category		
70–75	94 (48.5 %)	154,286 (42.6 %)^c^
76–80	58 (29.7 %)	95,894 (26.5 %)^c^
81–85	35 (17.9 %)	63,406 (17.5 %)^c^
86–90	6 (3.1 %)	34,358 (9.5 %)^c^
91 and over	2 (1.0 %)	13,811 (3.8 %)^c^
Marital status		
Married	107 (54.9 %)	170,560 (47.1 %)^c^
Single	24 (12.3 %)	55,371 (15.3 %)^c^
Widowed	58 (29.7 %)	125,551 (34.7 %)^c^
Divorced	3 (1.5 %)	3,767 (1.0 %)^c^
Separated	2 (1.0 %)	6,506 (1.8 %)^c^
GMS card holder	183 (93.4 %)	360,000 (96.0 %)^b^

*Abbreviations: OPTI-SCRIPT* OPTImizing PreSCRIbing for Older People in Primary Care, a cluster-randomised controlled trial, *GP* General Practitioner, *GMS* General Medical Services

*Sources:*

^a^O’Dowd et al. [51]

^b^Primary Care Reimbursement Service report 2011, Table 10 [52]

^c^Census of Population 2011

### Delivery to practices

All intervention practices received the same, standardised intervention. Practices participated in an academic detailing session with a research pharmacist in their own practice (lasting approximately 30 minutes). This involved a brief presentation on PIP and a practical component on how to use the web-based pharmaceutical treatment algorithms for the review of medications, using simulated patients, and a dummy username and password for the website. Each GP received Continuing Medical Education (CME) points for participating in the academic detailing session, which are necessary for ongoing registration with the national regulatory body. The academic detailing was described by the majority of GPs (9/10) as *“straightforward”* (GP16, intervention practice), and *“very useful”* (GP18, intervention practice). The research pharmacist reported that the GPs were receptive to the study objectives.

The control group practices were mailed simple feedback, outlining the participating patients and the particular category of PIP that applied to them. They were not encouraged to conduct medicines reviews or given any tools to support conducting reviews.

### Response of practices: how the intervention was adopted

#### Intervention group practices

Of the 11 practices in the intervention group, eight (73 %) conducted medicines reviews with the participating patients present web-based pharmaceutical treatment algorithms as outlined in the academic detailing (adoption as planned), two practices (18 %) conducted the reviews using the web-based pharmaceutical treatment algorithms in the absence of the patients and one practice (9 %) did not complete any medicines reviews. Where reviews were conducted without patients present (adaptation), GPs made notes in the patient charts regarding any changes to specified medication(s). One practice did not undertake face-to-face reviews with patients by choice. The second practice conducted the reviews without patients present due to study time constraints. Both were single-handed practices and the GPs were confident that their patients would accept the changes:

*“I think it’s probably easier in this practice because it is single-handed. Ok, you know, it’s not like I’m changing something that one of my colleagues put them on and said to them, you must stay on this or whatever, they all deal with me, for better or worse – I don’t know!”* (GP21, intervention practice).

While practices differed in terms of conducting reviews with and without patients present, all practices (10/10) were consistent in terms of a second adaptation and the use of the PILs. The PILs were not used as part of the review process in the intervention group. In the main, GPs forgot to provide patients with the PILs and indicated that in this patient group, such extra material was not necessary:

*“I didn’t have to, you know, the whole process is that our patients, if they trust us, and we explain everything to them, what we are doing, em, you don’t need to, we don’t need to do that* [give PILs]*.”* (GP16, intervention practice).

When asked if they would have liked to receive the PILs, generally this patient group reported not having much use for such materials:

*“Oh no, no, I don’t welcome those sorts of things; they just pile up here in the house.”* (P13.47, intervention patient).

The majority (9/10) of practices conducted reviews as patients presented for their repeat prescription, as recommended in the study protocol. However, one practice contacted the patients to attend a specific consultation outside of the repeat prescription timeline. Conducting the reviews with the patients was described positively by all GPs:

*“Oh yeah, it was very good, yeah, because actually, because they were coming in you were able to look at everything properly and they were coming in a structured review … just to give you a time to review the whole situation you know, in regards to all of their prescribing. It was very useful, yeah.”* (GP18, intervention practice).

The reviews were a positive experience for two main reasons. First, the intervention website and treatment algorithms were considered simple and easy to engage with by all GPs:

*“It was very straightforward, it worked well I thought, em it was clear and you know, from our point of view, actually when you actually got down to it, the patient, the actual process of going through the patient was quite quick.”* (GP24, intervention practice).

Second, the majority of GPs (8/10) reported that patients were overall *“very receptive”* (GP16, intervention practice) to the review process, making the consultation a positive and rewarding encounter from the GP perspective:

*“I actually think that this study has made me review my patients more closely, so I think it’s good for me personally, which means it’s good for my patients in the end.”* (GP23, intervention practice).

**Fig. 2:**
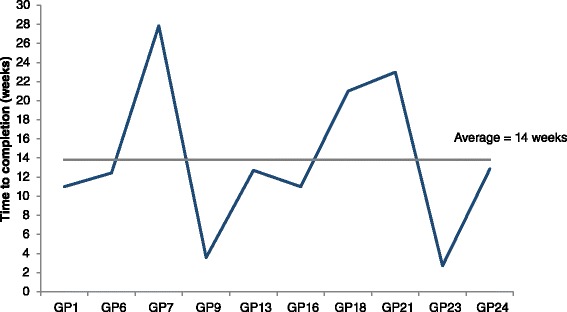
Time (weeks) from academic detailing to intervention practices completing all medicines reviews

Despite being asked to conduct all reviews within 6–8 weeks and receiving weekly reminder emails and calls, there were significant differences in the time between academic detailing and completion of all reviews by intervention practices (minimum 3 weeks, maximum 28 weeks) (Fig. 2). Longer completion times generally reflected a lack of resources to dedicate to the study within the practice:

*“That was difficult, because, the person who manages such things is* [practice nurse], *who was on sick leave for most of the study. So there was nobody driving the process because* [practice nurse] *was away, we had only very little nursing cover in her absence, then we were doing tasks that would have been previously done by the nurse, so it was a very busy time.”* (GP7, intervention practice).

#### Control group practices

The majority of the control group (80 %) reported that they did very little with the feedback letter provided to them. However, two GPs (20 %) reported that the simple feedback prompted them to change medications that may have been a concern to them (adaptation):

*“What I did was, I went in to all the files and I did a mail merge and wrote to them and changed their meds. So basically, there was a PPI - reduce the dose by half, so I just did that immediately and told them that I did that and why.”* (GP3, control practice).

### Recruitment and reach of individuals (patients)

Patient identification and recruitment was carried out by GP practices prior to practice randomisation as the study team did not have access to patient contact details or records until they consented to participate. This process was time-consuming and significant delays were introduced where GP practice staff were required to be involved (Appendix 4). Overall, patient identification and recruitment was reported as being quite onerous and was considered *“…the only graft”* (GP16, intervention practice) involved in participating in the study by many of the GPs. Recruitment burden was a source of frustration and annoyance for many of the GPs (7/10) and some had not anticipated the extra work it would involve:

*“I resented the reminders … I had underestimated the amount of involvement it would require from the practice. That’s what I would say.”* (GP7, intervention practice).

A total of 1306 patients were screened, 492 (38 %) of whom were found to be eligible and invited to participate. In total, 196 consented to participate, giving an overall response rate (enrolment fraction) of 40 %. Overall, six people needed to be screened in order to randomise one patient (Fig. 1). Recruited patients were demographically similar to the general population of those aged 70 and over in Ireland (Table 2).

### Delivery to individuals (patients)

#### Review outcomes

A total of 86 (87 %) reviews were conducted out of a potential 99; ten (10 %) were not conducted as one practice completed no reviews and three (3 %) were not conducted as the patient was deceased or withdrew. During the 86 medication reviews, 114 potentially inappropriate prescriptions were assessed. Of these, 44 (39 %) prescriptions were not altered, for reasons including the prescription being initiated in hospital (n = 10), patient preference (n = 9) and lack of available alternatives. The 62 remaining inappropriate prescriptions were altered, with the majority of changes (n = 30, 48 %) made in the form of a dose reduction, reflecting the fact that the majority of these changes were reductions in the use of proton pump inhibitors at maximum therapeutic dose.

Analysis of the OPTI-SCRIPT RCT data highlighted that the effectiveness of the intervention overall was largely mediated through a reduction in the prescribing of proton pump inhibitors with no statistically significant effect on other types of potentially inappropriate medicines such as long-term use of benzodiazepines. Some GPs were sceptical about the benefits of discontinuation of such medications in older patients:

*“Sometimes, for example, in relation to benzodiazepine, em, you know, somebody might be on benzodiazepines and has been for 40 years, which one of the patients actually was, I don’t think it’s appropriate to stop that. If they’re stable and they can get on with their lives then I think it would cause more hassle for them.”* (GP1, intervention practice).

However, out of 14 prescriptions of long-term benzodiazepine in the intervention group, five (36 %) were altered while the remaining nine (64 %) were unaltered due to patient preference.

**Fig. 3:**
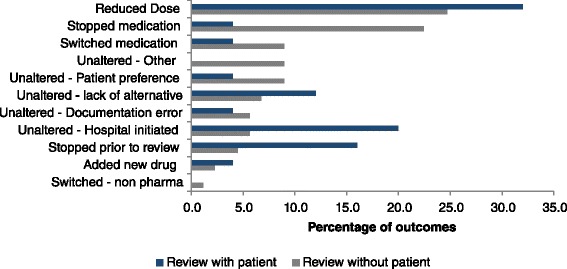
Comparison of changes to PIP drugs during medicines reviews conducted with and without patients present

Of the reviews conducted, 67 (78 %) were conducted with the patient present and 19 (22 %) were conducted without the patient present. A total of 89 potentially inappropriate prescriptions were assessed during reviews with patients present compared to 25 potentially inappropriate prescriptions in reviews conducted without patients present. Although the numbers are small, differences in the changes made to PIP drugs during reviews conducted with and without patients present were observed (Fig. 3) with a higher proportion of reviews conducted with patients present resulting in a complete removal of the medication (22 %) compared to reviews without the patients (4 %). Switching to alternative, more appropriate medications was also more common when the patient was present at the review (9 % compared to 4 %). Practices who conducted reviews without the patient present were all single-handed practices.

### Response of individuals (patients) to the medicines review process

All patients interviewed reported that medicines reviews were a good idea and regardless of whether or not changes were made to their medication regimen, patients were receptive to the idea as *“nobody wants to take more medications than they need to”.* (P13.45, intervention patient).

Overall, patients’ responses to the medication review process were classified across the themes of benefits and barriers (Table 3). Benefits of the reviews included a perception of receiving high-quality care for the majority of patients (7/11), providing reassurance that their health and well-being was a priority. The review provided an opportunity for patients to examine their medications as many (6/11) recognised the potential to be taking medications that may no longer be necessary. Two patients reflected on the wider societal implications of taking medications that may no longer be clinically necessary as wasteful. Both highlighted that by reducing waste in this area, there may be the potential to save money that could be redistributed in other areas. However, despite the overall positive views on the medication reviews, a number of patients highlighted that GP time and workload were a barrier (Table 3).

**Table 3:** OPTI-SCRIPT process evaluation: themes and supporting quotes

Main theme	Sub-theme	Example quote
Delivery to practices	Academic detailing quality	“Yes, that was very informative, very straightforward, very user friendly.” (GP16, intervention practice)
Response of intervention group	Adoption as planned	“O yeah, it was very good, yeah, because actually, because they were coming in you were able to look at everything properly and they were coming in a structured review .... .just to give you a time to review the whole situation you know, in regards to all of their prescribing. It was very useful, yeah.” (GP18, intervention practice).
	Adaptation I. Reviews without patients	“So I didn’t do it with the patients but what I did was, I think you saw from the patient records, I highlighted the notes on it, and I’d have put tags on charts when I found, yeah that needs to be done, to be addressed with their next prescription. (GP21, intervention practice).
	II. Patient information leaflet non-use	“I didn’t have to, you know, the whole process is that our patients, if they trust us, and we explain everything to them, what we are doing, em, you don’t need to, we don’t need to do that [give PILs].” (GP16, intervention practice).
	Facilitators of implementation I. Simplicity	“It was very straightforward, it worked well I thought, em it was clear and you know, from our point of view, actually when you actually got down to it, the patient, the actual process of going through the patient was quite quick.” (GP24, intervention practice).
	II. Patient receptivity	“Absolutely no problem at all. And in fact, if anything they were quite glad, you know, that somebody is looking at their medications and making sure that it is OK, and all the rest.” (GP1, intervention practice).
	Barriers of implementation I. Staff	“That was difficult, because, the person who manages such things is [practice nurse], who was on sick leave for most of the study. So there was nobody driving the process because [practice nurse] was away, we had only very little nursing cover in her absence, then we were doing tasks that would have been previously done by the nurse, so it was a very busy time.” (GP7, intervention practice).
Response of control group	Adaptation	“What I did was, I went in to all the files and I did a mail merge and wrote to them and changed their meds. So basically, there was a PPI - reduce the dose by half, so I just did that immediately and told them that I did that and why.” (GP3, control practice).
Recruitment and reach in individuals	Recruitment burden	“I resented the reminders … I had underestimated the amount of involvement it would require from the practice. That’s what I would say” (GP7, intervention practice)
Responses of individuals	Benefits of reviews I. Quality of care	“I think it is important really, because it makes people feel, well, you know that there is somebody that cares. You know, as you are getting older, that there is somebody that cares about the elderly, that they, you know, are being properly looked after and people are thinking about them.” P23.38
	II. Societal good	“I’m sure, I’m absolutely sure, there through not the patient’s fault, eh, there must be an amazing amount of pharmaceutical waste consumed by patients who don’t really eh, need it. And as you say, the purpose of your exercise is to find out if some of these can be dropped. In fact, I’m sure they could be and, the monies saved by the State could eh, be put into looking after the less fortunate people.” P1.61
	III. Necessary medication	“You’re inclined to go on things and be left on them and then you wonder should you be on them all that time, is there any side effects with them, all that kinda thing.” P7.4
	Barriers to reviews I. GP workload	“Well, I mean, if my, if my GP has time to do that sort of thing then fine, you know.” P18.48
Future implementation	Facilitators I. Positive aspiration	“When you are a GP you get practices and you get bad habits, and you get good habits, and sometimes you are too busy to change your habits until it is pointed out so, anything like this is a good thing.” (GP19, control practice).
	II. Cardinal PIPs	“I think that if you keep it simple, and maybe in a structured way if you could layer it, so that you know, for 2012 we are focusing on these five issues and in 2013 we’re focusing on these, you know. There would be a little bit of slippage with last year’s issues, but over time you would introduce better prescribing.” (GP13, intervention practice).
	Barriers I. Workload	“General practice at the moment now, as far as I can see, is getting hit by about 30 % more extra work, due to the economic downturn, so most medical card list have gone up by about 30 %, and that is increasing a huge volume of work, because those patients before, happened to be in the non-medical card area and they weren’t consulting as much. So they are now consulting, eh, much more frequently so it’s very little time left … if you had to do that every 6 months, to review all those patients. Where would you get the time?” (GP5, control practice).
	II. Reimbursement	“I often wonder if the government was to pay a fee for us to review ten patients every 3 months formally, but they’re going to say, we’re already paying you to do these prescriptions, to write these prescriptions you know, like come on guys, and they are right.” (GP13, intervention practice).

### Future implementation: GP perspective

The future implementation of an intervention to assist with conducting medication reviews was mainly viewed as a positive aspiration. GPs from both intervention and control groups expressed a desire to learn and a willingness to change their prescribing practices:

*“When you are a GP you get practices and you get bad habits, and you get good habits, and sometimes you are too busy to change your habits until it is pointed out so, anything like this is a good thing.”* (GP19, control practice).

Patients also perceived the value of the objectives of the medication review process and expressed support for the opportunity to stop unnecessary medications. Despite this enthusiasm, GPs did not seem to consider this as part of their current core work, and a number of organisational barriers to the provision of medicines reviews as standard in Irish primary care in the future were identified, particularly in relation to workload and reimbursement.

GPs highlighted that current workloads made dedicated reviews for all older patients unfeasible due to time constraints:

*“General practice at the moment now, as far as I can see, is getting hit by about 30 % more extra work, due to the economic downturn, so most medical card list have gone up by about 30 %, and that is increasing a huge volume of work, because those patients before, happened to be in the non-medical card area and they weren’t consulting as much. So they are now consulting, eh, much more frequently so it’s very little time left … if you had to do that every 6 months, to review all those patients. Where would you get the time?”* (GP5 control practice).

Solutions offered by GPs to this workload problem included the use of alerts embedded into practice management systems and involving pharmacists in the medication review process. A number of GPs highlighted that focusing on a select number of high-risk or “cardinal PIPs” would make the process more manageable:

*“I think that if you keep it simple, and maybe in a structured way if you could layer it, so that you know, for 2012 we are focusing on these five issues and in 2013 we’re focusing on these, you know. There would be a little bit of slippage with last year’s issues, but over time you would introduce better prescribing*.” (GP13, intervention practice).

Reimbursement within the current structures was also highlighted as a barrier. At present, the majority of patients aged 70 years and older are General Medical Services (GMS) patients and as such, access the GP free at the point of use. GPs are reimbursed via capitation by the state. GPs highlighted that there may be resistance from both patients and the government to paying extra for a medicines review consultation.

*“I often wonder if the government was to pay a fee for us to review ten patients every 3 months formally, but they’re going to say, we’re already paying you to do these prescriptions, to write these prescriptions you know, like come on guys, and they are right.”* (GP13, *intervention practice*).

*“Unless it’s free they won’t come in and even if it is free, I don’t know, it’s difficult to get them in you know. Em, if they are paying, definitely they won’t want to come in to do something that they think is for your benefit and not for theirs you know.”* (GP18, intervention practice).

## Discussion

This process evaluation combined qualitative and quantitative methods to enhance understanding of the implementation of the OPTI-SCRIPT intervention, the experiences of those participating in the study, and lessons for future implementation.

### Intervention implementation

The results demonstrate that there was variation in how the intervention components were delivered by GP practices despite receiving a standardised academic detailing session. First, just over 70 % of practices completed medicines review as recommended with the patient present. Medications were more likely to be completely stopped or switched to another more appropriate medication when reviews were conducted with patients present. Organisational factors such as resources and workload are often reasons for variation in intervention delivery [24]. In this case, practice characteristics may have been influential as both practices who conducted reviews without patients present were single-handed practices. Strong evidence supports the active involvement of older people in their primary care episodes to improve health outcomes [25, 26]. While the effects of patient participation in medication reviews are understudied [27], recent evidence from the EMPOWER study highlights that increasing older people’s participation in prescribing decisions can result in the discontinuation of inappropriate benzodiazepines (risk difference 23 %, 95 % CI 14–32 %) [28]. Although based on small numbers, our findings, and those of the EMPOWER study, provide support for actively encouraging shared decision-making between older patients and primary care prescribers, even for medications typically considered difficult to change such as benzodiazepines. It could be argued that medications such as proton pump inhibitors are easier to alter where time and resources are limited but the fact that 36 % of benzodiazepines were altered in the OPTI-SCRIPT study would suggest otherwise. Second, the PILs were not utilised by participating GPs. Evidence indicates that PILs are promising tools in reducing antibiotic prescribing in primary care and that older patients appreciate being provided with brief, clearly written information leaflets in addition to information from their doctor [29, 30]. Both GP and patient participants in this study felt that PILs were unnecessary, however, as the GPs reported forgetting this element of the intervention, it could be argued that its importance was not fully conveyed during the academic detailing process.

The control group also varied in its behaviour, with two practices implementing changes based on the feedback letter they received – a common finding in prescribing-based RCTs [31]. This activity was the driving force for the changes noted in the control group in the RCT analysis. The fact that only a small proportion of the control group implemented changes reinforces the evidence that less intensive feedback on prescribing behaviour is generally not sufficient to impact on prescribing practices [32, 33].

Overall, these findings are consistent with previous studies that have highlighted that complex interventions in primary care are often not implemented and utilised as intended [34, 35].

### Participant experience

Both GP (32 %) and patient (40 %) recruitment rates were modest. The rates compare favourably to similar PIP related RCTs [36], but smaller than reported in other primary care studies [37]. For those GPs and patients who did take part, participation in the OPTI-SCRIPT study was positively viewed. The intervention was considered simple to engage with and the participants reported to agree in principle with the overall study objectives (i.e. stopping or reducing unnecessary medications in older patients). The OPTI-SCRIPT study also provided the opportunity for altruism, and perceived greater quality of care, important factors influencing the participation of older people in RCTs [38]. For GPs, the study provided the opportunity to improve care for current patients, and update their knowledge and clinical skills, commonly reported benefits of participation in research [39–41]. Volunteer bias may also provide an explanation for the overall positive experiences reported. Those who volunteer for research studies may be motivated by a particular interest in the study objective. Comparison with a national sample of GPs highlighted that OPTI-SCRIPT GPs may have been more research-orientated but OPTI-SCRIPT patients’ demographics reflected those of the general population.

GPs’ main critique of study participation related to patient recruitment, which is consistently one of the most challenging aspects of research studies. The process of identification and recruitment required more time than expected, consistent with previous trials recruiting older patients in primary care [42]. The numbers needed to screen was six, meaning six people needed to be screened in order to randomise one, larger than has been reported in other primary care based studies (median 2.43 [37]). Workload and time remain significant barriers to participation in such research by GPs. It is important to minimise the efforts required by practice staff to recruit patients [43]. In the UK, research networks such as The Clinical Research Network offer services to assist with timely recruitment, however, in this case, no such services operate and ethical committee requirements were such that the burden of recruitment fell heavily on the practices involved. Excessive delays, variability in process and outcome, and imposed requirements that can have negative consequences for study conduct are common challenges reported during the research ethics review process for cluster RCTs [44]. In the absence of support services, electronic identification of potential participants could potentially expedite the recruitment process.

### Future implementation

The main RCT demonstrated that the OPTI-SCRIPT intervention was effective in reducing PIP in primary care [12]. The challenge remains how to embed this type of intervention within the current structures of health care services. Rogers (2003) identified the key attributes (as perceived by prospective adopters), that influence the adoption of an intervention into practice [45]. Innovations that are perceived as having greater relative advantage (innovation is perceived as better than the idea it supersedes), compatibility (agreement between the innovation and organizational values and beliefs), simplicity (degree of difficult to use), trialability (how much innovation can be experimented with), and observability (ease with which results can be seen) will be adopted more rapidly than others. The process evaluation identified OPTI-SCRIPT as having relative advantage, compatibility, observability and was low in complexity. Coupled with the predominantly positive experiences reported during the process evaluation, this would suggest that the implementation of such an intervention would be acceptable to both GP and patient participants alike. However, medicines reviews did not seem to be considered core work. Despite the enthusiasm expressed, there was considerable variation in the time taken to complete the reviews (3–28 weeks) and reminders from the study team were required. In order to implement medicines reviews as standard practice outside of the study setting, the organisational barriers of GP workloads, time to conduct the reviews, and reimbursement mechanisms need to be considered. Focusing on a select number of specific medications or “cardinal PIPs” offers a means to decrease workload barriers.

Just under 40 % of potentially inappropriate prescriptions were unaltered due to factors such as hospital initiation, patient preference and lack of alternatives, serving as a reminder that although a medication may be considered inappropriate for older people, it may still be prescribed [46].

### Strengths and limitations of this study

This study is one of a growing number of process evaluations published independently of the main RCT findings [15, 47, 48] and is one of first to adopt a process evaluation framework explicitly intended for use in cluster RCTs [19]. Quantitative and qualitative data were systematically collected and rigorously analysed, providing an insight into patient and GP perspectives of the intervention and its implementation.

A number of important findings emerged from the qualitative data which would not have been evident from the quantitative analyses such as the variation in conducting reviews with and without patients present, reinforcing the important contribution that qualitative methods can have in such evaluations [49]. However, the limitations of this study lie primarily with the collection of qualitative data as some participants were not available for interview. In particular, the GP who did not conduct any of the medication reviews was not interviewed. The practice provided the OPTI-SCRIPT team access to patient data, but attempts to schedule an interview with the GP were unsuccessful. As the only GP not to complete the reviews, this interview would have provided some valuable insights. The GP indicated that although the practice intended to conduct the reviews, they had no time to complete the study. A further limitation to the qualitative data may be the length of the interviews which were an average of 15 minutes with GPs and 12 minutes with patients which may have affected the richness of the data collected; however, all items on the topic guide were addressed.

### Lessons learnt and future research

The valuable insights gained during this process evaluation are summarised in Table 4. Based on these findings, revisions to the intervention will be made and a definitive trial with a larger sample size and longer follow-up period will be conducted.

**Table 4:** Insights/key messages from the OPTI-SCRIPT process evaluation

• Complex interventions in primary care are often not implemented and utilised as intended. • Intervention delivery may vary by practice characteristics such as number of GPs and practice resources. • Recruitment continues to be one of the most challenging aspects of conducting trials in primary care. In this setting, computerisation of patient identification would decrease the requirements placed on GPs at the start of the study and speed up the recruitment process. • Involving patients in medication reviews has the potential to decrease inappropriate prescribing. • Targeting a smaller number of specific medication groups or “cardinal PIPs” emerged as an important facilitator in overcoming workload barriers. • Process evaluations are more informative when they incorporate both qualitative and quantitative research methods.	

## Conclusions

In summary, decreasing PIP in primary care is achievable, particularly through involving older patients in medication reviews. The OPTI-SCRIPT RCT and process evaluation found that the intervention was effective, feasible and was acceptable to GPs and older patients. However, PILs were not a successful component of the intervention. Plans for wider implementation of the intervention in primary care would need to increase support for patient recruitment and address organisational barriers to implementation such as lack of time in busy GP practices and remuneration.

## Abbreviations

CME, continuing medical education; GMS, General Medical Services; GP, General Practitioner; HRB, Health Research Board; ICGP, Irish College of General Practitioners; OPTI-SCRIPT, OPTImizing PreSCRIbing for Older People in Primary Care, a clusTer randomised controlled trial; PILs, patient information leaflets; PIP, potentially inappropriate prescribing; RCT, randomised controlled trial

## Figures and Tables

**Fig. 4:**
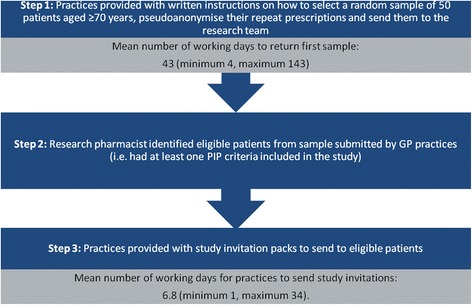
Patient identification and recruitment process

## Appendix 1

**Table 5:** Overview of the OPTI-SCRIPT cluster RCT methods and findings [11]

Aim	To test the effectiveness of a multifaceted intervention in reducing the level of PIP in primary care.
Participants	Twenty-one general practices (intervention n = 11, control n = 10). A total of 196 patients ≥70 years (intervention n = 99, control group n = 97) with ≥1 PIP drugs.
Outcome measure	Proportion of participant patients with PIP and the mean number of potentially inappropriate prescriptions per group.
Intervention group	The intervention consisted of: (1) Academic detailing with a pharmacist One session (lasting 30 minutes) where a pharmacist visited the practice to discuss PIP, medicines review and the web-based pharmaceutical treatment algorithms (2) Medicines review with web-based pharmaceutical treatment algorithms. GPs were asked to conduct one review per patient using the web-based platform to guide them through the process. The GP was presented with the specific PIP drug(s) for each patient, and for each PIP drug, there was a treatment algorithm with the following structure: a. The individual PIP with reason for concern b. Alternative pharmacological and non-pharmacological treatment options c. Background information (where relevant) 3) Patient information leaflets to give to patients during the review. Each leaflet: a. Described the PIP and the reasons as to why it may be inappropriate b. Outlined the alternative pharmacological and non-pharmacological therapies GPs may offer
Control group	Control practices delivered usual care. Usual care for public General Medical Services (GMS) patients allows GPs to give a prescription on a monthly or 3 monthly basis. Control practices received simple patient-level PIP feedback in the form of a list summarizing the medication class to which the individual patient’s potentially inappropriate medication belonged. Control practices did not receive an academic detailing visit or were not prompted to carry out medicines review with the individual patients.
Results	Upon intervention completion: • OPTI-SCRIPT intervention group had significantly lower odds of having PIP than control group (adjusted OR 0.32, 95 % CI 0.15 to 0.70, *P* < 0.01). • Mean number of PIP drugs in the intervention group was 0.70, compared to 1.18 in the control group (*P* < 0.01). • The intervention was effective in reducing proton pump inhibitor prescribing (adjusted OR 0.30, 95 % CI 0.14 to 0.68, *P* = 0.04).

*Abbreviations: OPTI-SCRIPT* OPTImizing PreSCRIbing for Older People in Primary Care, a clusTer randomised controlled trial, *RCT* randomised controlled trial, *CI* confidence interval, *GMS* General Medical Services, *PIP* potentially inappropriate prescribing, *OR* odds ratio

## Appendix 2

**Table 6:** Key features of Irish Primary Care

• Mixed public private funding. • No national register of GPs is in operation, but it is estimated that there are approximately 2500 GPs in Ireland. • Three categories of eligibility to primary health care: ○ Full eligibility: free access to primary health care via the General Medical Services (GMS) scheme, which is means tested. Prescription co-payments were introduced in 2010, and amount to €2.50 per item, up to a maximum of €25 per family in 2014. ○ Limited eligibility: free access to GP visits but are required to pay for all prescriptions up to a monthly limit of €144 per family. ○ Private patients: non-GMS patients are required to pay in full for primary care services (approx. €50 per GP visit) and are entitled to limited free public health services such as maternity services and childhood immunisations. Prescription costs are paid in full up to a monthly limit of €144 per family. • An estimated 97 % of people aged 70 and over qualify for the GMS scheme. • Standardised medication reviews for community-dwelling older patients are not specifically recommended as is the case in the UK with the National Service Framework for Older People.	

## Appendix 3

### Topic guide

Topic guide: GP

Broad prescribing-related interview questions

Could you tell me a little about your experience of prescribing for your older patients?

*Prompts:* multimorbidity; polypharmacy; patient preference/demands

Are you familiar with the concept of PIP or the criteria used to measure it, aside from this study?

Could you tell me a little about your perspective on PIP? What is your view on PIP in primary care?

In your opinion, how is PIP important, relevant to practice?

What is your opinion of the terminology used, PIP?

*PIP as a concept*

Are you familiar with the concept of PIP or the criteria used to measure it, aside from this study?

Feelings about the concept in general:

- Could you tell me a little about your perspective on PIP? What is your view on PIP in primary care?
- Feelings re the terminology
- In your opinion, how is it important, relevant to practice?

***For intervention GPs only***

You had the AD session with the pharmacist and then conducted the reviews with the patients

- What did you think of the AD session overall?
  - ○ Trainer
  - ○ Content
  - ○ Information
  - ○ Length
- Conducting the reviews – how do you feel the process worked?
- Elements that worked
  - ○ How was information incorporated in routine practice?
  - ○ How was helpful/unhelpful?
  - ○ Information system, website, algorithm?
  - ○ How did patients respond?
    - ▪ Did you give them leaflets – feelings about them?
- Elements that may benefit from change
  - ○ What was not helpful
  - ○ Are there specific barriers to changes?
  - ○ What could be done better?
- What would the ideal process look like to you?
- Can you comment on the time needed for this?
  - ○ Longer than general consult, much preparation involved?
- Making changes
  - ○ Role of patient
  - ○ Role of hospital
- Effects of intervention overall
- Overall, what was your experience of taking part in this study?

***For control GPs only***

So now I would like to talk to you about what you did during the study. We sent you a list, of the participating patients with an ID number of drug class where there may be a concern.

- What did you do with this information?
  **Table:**
  Nothing • Can I ask what you thought of the information i.e. – vague, not useful etc • What would have made it more useful to you i.e. – more detail on exact issue, alternative advice etc?	Something • Details of what they did • Why that course of action? • Would it be possible/feasible to do this for all their patients?
- What would be helpful for you?
- What would the ideal process look like to you?
- Overall, what was your experience of taking part in this study?

### Concluding comments

- Anything participant would like to add/any questions for me?

**Table:**

Probes: In what way? Can I ask why/how? Really?	

### Topic guide – patient

Broad medication-related interview questions

Could you tell me a little about your medications?

*Prompts:* what do you take; how many medicines; are your medicines important/necessary; why do you take them; are there any side effects?

Could you tell me how you feel about taking these medications?

*Prompts:* how does having to take your medicines impact on you?

Could you tell me a little about your interactions with your GP?

*Prompts*: how often do you see them; what do you discuss about your medications; what type of information about your medications do you get?

The intervention - you met with your GP to discuss some of your medications in a medicines review

- Can you tell me how you found that experience?
  - ○ Informative, beneficial, helpful/unhelpful, negative
    - Can you tell me more about why you feel that?
  - ○ How did it differ from your general GP visits?
    - ▪ Get anything extra from it (PIL)?
      - Yes – was it useful/informative
      - No – would you like something like this?
  - ○ What was the outcome if you don’t mind discussing it?
    - Did anything change (e.g. stopped medication, reduced dose)?
      **Table:**
      YES • How do you feel about those changes – were they positive, what impact have they had on you/health/life? • Are you happy?	NO • What was the reason for that – your preference, hospital, no alternative? • Are you happy with the outcome generally?
- Do you think having a review like this, maybe once or twice a year would be useful?
  - ○ To you, to others etc.
- Elements that may benefit from change
  - ○ Was there anything you disliked – a process you would like to change?
- How would you describe the experience of taking part in this study?
  - ○ Positive, negative, useful, neutral etc.
- Effects of intervention overall
  - ▪ In summary, given your experience of the review, is it something you would be happy to do again (hypothetically) if your GP asked, would you like to see it become a routine thing?

## Footnotes

1 All practice and patient baseline data was collected prior to minimisation of practices to minimise selection bias.
